# Dietary Iodine Intake of the Australian Population after Introduction of a Mandatory Iodine Fortification Programme

**DOI:** 10.3390/nu8110701

**Published:** 2016-11-04

**Authors:** Karen Charlton, Yasmine Probst, Gabriella Kiene

**Affiliations:** 1 School of Medicine, Faculty of Science, Medicine and Health, University of Wollongong, Wollongong 2522, NSW, Australia; gk375@uowmail.edu.au; 2 Illawarra Health and Medical Research Institute, Wollongong 2522, NSW, Australia; yasmine@uow.edu.au; 3 Smart Foods Centre, School of Medicine, Faculty of Science, Medicine and Health, University of Wollongong, Wollongong 2522, NSW, Australia

**Keywords:** iodine, fortification, Australia, dietary intake, bread, monitoring

## Abstract

To address mild iodine deficiency in Australia, a mandatory fortification program of iodised salt in bread was implemented in 2009. This study aimed to determine factors associated with achieving an adequate dietary iodine intake in the Australian population post-fortification, and to assess whether bread consumption patterns affect iodine intake in high-risk groups. Using nationally representative data of repeated 24-h dietary recalls from the 2011–2012 Australian National Nutrition and Physical Activity Survey, dietary iodine intakes and food group contributions were compared by age, socioeconomic status (SES), and geographical remoteness (*N* = 7735). The association between fortified bread intake and adequacy of iodine intake (meeting age and sex-specific Estimated Average Requirements) was investigated using logistic regression models in women of childbearing age 14–50 years (*n* = 3496) and children aged 2–18 years (*n* = 1772). The effect of SES on bread consumption was further investigated in a sub group of children aged 5–9 years (*n* = 488). Main sources of iodine intake at the time of the survey were cereal and cereal products, followed by milk products and dishes. Differences in iodine intake and dietary iodine habits according to age, SES and location were found (*p* < 0.001) for women of child-bearing age. Fortified bread consumption at ≥100 g/day was associated with five times greater odds of achieving an adequate iodine intake (OR 5.0, 95% CI 4.96–5.13; *p* < 0.001) compared to lower bread consumption in women and 12 times in children (OR 12.34, 95% CI 1.71–89.26; *p* < 0.001). Disparities in dietary iodine intake exist within sectors of the Australian population, even after mandatory fortification of a staple food. On-going monitoring and surveillance of iodine status is required.

## 1. Introduction

Iodine deficiency is the largest preventable cause of brain damage and mental impairment worldwide. Iodine is required for thyroid hormone production, which is central to metabolism and growth through the lifecycle. As well as irreversible mental retardation in its most severe form, iodine deficiency can also result in miscarriages, stillbirths, and impaired psychomotor development and behavioural problems in children born to iodine deficient mothers [1] Australia was identified as a country with mild iodine deficiency, based on urinary iodine concentrations identified in school children aged 8–10 years across five mainland states in the Australian National Iodine Nutrition Study, 2003–2004 [2] as well as smaller, non-representative studies of pregnant and lactating women [3,4,5,6]. To address the re-emergence of iodine deficiency in Australia [7], the government introduced mandatory iodine fortification of salt used in bread in 2009 [8]. The level of iodine required by mandatory fortification to be added to salt used in the bread-making process was modelled using bread consumption patterns of 100 g/day, equating to approximately three slices [8]. However, dietary and food composition data at that time was outdated [9] and current patterns of bread intake were unknown. In a small convenience sample in Victoria, median bread intake in pregnant women was reported to be only two slices per day, while less than half (43%) of participants ate three or more slices per day [10]. Despite the mandatory iodine fortification policy, high-risk groups with increased requirements, including pregnant and lactating women, may still not be adequately protected [11]. Dietary studies report an increased iodine intake in pregnant women since the implementation of fortification [12,13,14]. But these studies were not conducted in nationally representative samples of the population. Young children are also vulnerable to inadequate iodine intakes because of their less varied dietary exposure and importance of the nutrient for growth and development. The International Council for the Control of Iodine Deficiency Disorders (ICCIDD) recommends that primary school children aged 6–12 years are the universal reference group to be used as an indicator of population-level iodine status. Children of this age are targeted for collection of spot urine samples for determination of median urinary iodine concentrations (UIC) which are compared against reference values of 100–199 µg/L that suggest adequacy of intake.

Studies have shown a socioeconomic gradient for micronutrient intakes, with low socioeconomic status (SES) groups more likely to have poor micronutrient intakes compared with their higher SES counterparts [15,16,17]. In Australia, low SES groups are less likely to comply with dietary guidelines compared with higher SES groups. Despite sparse information about dietary iodine intake in Australian pregnant women [18], low SES groups have been documented to be less likely to consume recommended iodine supplements during pregnancy as compared with higher SES groups [19], as reported in other countries [15,16,17,18]. Low SES groups are also less likely to comply with dietary guidelines when compared with higher SES groups [20] and have various barriers to healthy eating which affects nutrient intakes, including high costs [20], lack of priority for health [17], difficulties accessing healthy foods [21], and a lack of social support [22]. Previous investigations into iodine deficiency have speculated that the high cost of iodine-rich food sources such as fish and seafood, as well as a lack of knowledge, low uptake of iodine supplements, and taste preferences might be major barriers to achieving adequate iodine intake in pregnant women [3,23].

Ensuring adequate iodine intake amongst women of childbearing-age is important, in order to ensure optimal iodine intake during early pregnancy and to avoid disruption to foetal brain growth and body development [11]. The most recent national dietary survey in Australia, the National Nutrition and Physical Activity Survey (NNPAS), conducted in 2011–2012 did not sample pregnant or breastfeeding women, therefore this study will instead draw primarily on data for women of childbearing age (14–50 years).

Currently, in Australia no studies have determined contributions of food sources, including fortified bread, to iodine intake across life stages and by sociodemographic factors in a nationally representative sample. Further to this, assessing at risk groups such as women of childbearing age and children who have been previously been found to have inadequate iodine status is necessary to evaluate the effectiveness of the fortification program in eradicating iodine deficiency. This information is required to inform the development of dietary guidance related to achieving and optimal iodine intake. The aims of the present study were: (1) to determine sociodemographic factors associated with achieving an adequate dietary iodine intake post-fortification in the Australian population as a whole, and to identify food group contributions to total dietary iodine intake; (2) to assess whether bread consumption patterns (≥100 g bread/day) affect iodine status in high risk groups (women of childbearing age, 14–50 years; and children, 2–18 years), after adjusting for high iodine food sources, SES, geographical location and age; and (3) to determine the effect of age, and SES on bread consumption patterns and iodine intake in primary school children aged 5–9 years.

## 2. Materials and Methods

### 2.1. National Nutrition and Physical Activity Survey Dataset

A secondary analysis of the NNPAS 2011–2012 was utilised for this study. Detailed information about the NNPAS methods have been published elsewhere [24]. Briefly, the NNPAS was conducted between May 2011 and June 2012 using a sub-sample of respondents from the Australian Health Survey (AHS) 2011–2013. The AHS was conducted across all states of the Australian continent with the intent to create a representative sample of the Australian population. The NNPAS was a cross-sectional survey designed to obtain information on both food and nutrient intakes and physical activity patterns of the Australian population. The survey provides detailed data on food and nutrient intakes, food habits, food security, tobacco smoking, physical activity behaviours and selected physical measurements [24].

#### 2.1.1. Sampling

The NNPAS used a stratified multistage area sample design from which a sample of private dwellings in urban and rural areas across all states and territories of Australia were randomly selected. Within selected dwellings, a random sub-sample of residents was selected including one adult (18 years and older) and where applicable one child (2–17 years). Households were selected from each state and territory proportional to the stratum specific population in that state/territory. The area-based selection ensured that all sections of the population living in private dwellings within the geographic scope of the survey were represented by the sample. The final response rate was 77% resulting in a nationally representative sample size of 12,153 persons (≥2 years) from 9519 households throughout Australia [24].

#### 2.1.2. Dietary Assessment

Computer-assisted personal interviewer-administered 24-h dietary recall dietary assessments were collected, as described by the Australian Bureau of Statistics (ABS), using a food model booklet to estimate portion sizes [25]. The primary caregiver provided the dietary recall for children aged 2–8 years and while children aged 9–17 years self-reported their intake. A second 24-h computer-assisted telephone-administered interview (CATI) administered dietary recall was completed by 64% (*n* = 7735) of participants at least eight days after the first.

Population weights based on age, gender and region and computed by the ABS were applied for all analyses to account for the non-proportional sampling scheme of the survey and to reflect the population at the time of the survey.

#### 2.1.3. Nutrient and Food Group Analysis

Reported dietary intakes of the NNPAS participants were translated into nutrient intakes by Food Standards Australia New Zealand using the AUSNUT 2011–2013 food composition database [26]. For this study, dietary iodine intake was determined using an average of the two 24-h dietary recalls (*n* = 7735) in an attempt to reduce within-person variation. Independent t-tests were conducted to determine whether differences existed between estimated iodine, calculated from a single 24-h recall and the average of repeated 24-h recalls. A significant difference (*p* < 0.05) was found between iodine intake using a single compared to an average of two days of repeated 24-h recalls. Therefore, only participants who completed both the first and second 24-h dietary recall were included in the current analyses. Foods were classified into a nested hierarchical food grouping system of major, sub-major and minor food groups [27] and the contribution of food groups to iodine intake was determined.

For the analyses related to adequacy of iodine intake according to bread consumption of ≥100 g bread per day, a sub-sample of women of child-bearing age and children (2–18 years) with repeated 24 h dietary recalls were included in this study (*n* = 3496 and 1772, respectively). Sub group analyses of the effect of SES and geographical area of residence on bread consumption patterns and total iodine intake were conducted in a subsample of children aged 5–9 years (*n* = 488). Bread intake was determined using the definition provided by the Food Standards Code Australia New Zealand for the iodine fortification program [28] as any product made by baking yeast-leavened dough prepared from one or more cereal flours or meals and water. Sub-major food groups considered to relate to this definition were: Regular bread, and bread rolls (plain/unfilled/untopped varieties); flat breads (e.g., pita bread, naan bread); focaccia and pide (Turkish bread); bagels (white, wholemeal, sweet); topped breads, buns and rolls (e.g., cheese and bacon rolls); baked English-style muffins (white, white high fibre, multigrain, wholemeal, and fruit); sweet buns; and fruit breads and rolls (e.g., raisin bread). Chemically aerated non-yeast leavened dough, such as damper and soda breads, that are exempt from this definition of bread and do not require the use of iodised salt were excluded from the bread category for the purpose of this analysis.

#### 2.1.4. Determining Socioeconomic Status and Geographical Location

The SES of participants was determined using the Index of Socio-economic Disadvantage (IRSD), based on the area of residence of the participant. The IRSD provides a measure of SES in a census collection district based on data from the most recent 2011 census data. IRSD is part of the Socio-Economic Indexes for Areas 2011 [29] classification and the index includes variables such as income, educational attainment, unemployment, and dwellings without motor vehicles. The lowest Socioeconomic Index For Areas (SEIFA) quintile captures the most disadvantaged areas, while the highest quintile represents the most advantaged areas.

Geographical location of participants were categorised using the Australian statistical geography standard 2011 Accessibility/Remoteness Index of Australia (ARIA) [30]. The remoteness structure divides each state and territory into several regions on the basis of their relative access to services. Three classes of remoteness were used based on the census district of residence at the time of the survey [30].

### 2.2. Statistical Analyses

For the entire sample (*n* = 7735), non-parametric analyses were used for iodine intakes and food group contributions, as assessed by Kolmogorov-Smirnov distribution testing (*p* < 0.001). The Kruskal-Wallis ANOVA was performed to examine whether iodine intake and contributing food groups differed between age groups (2–3; 4–8; 9–13; 14–19; 19–30; 31–50; 51–70; 71+ years) for adequacy of intake based on Nutrient Reference Values [31], geographical remoteness areas (major cities, inner regional and other regions) and quintiles of relative socio-economic disadvantage. Pairwise comparisons were performed for all significant results from Kruskal-Wallis tests, using Dunn’s procedure [32] with Bonferroni correction for multiple comparisons; adjusted *p*-values are presented. Due to the large sample size, effect size was measured for all analyses using eta-squared method and Cohen’s guidelines were used to determine effect size (0.1 = small effect; 0.3 = median effect; 0.5 = large effect) [33].

Prevalence of adequacy of iodine intake by bread intake group was investigated using logistic regression models for women of childbearing age (*n* = 3496) and all children aged 2–18 years (*n* = 1772). Mean 2-day iodine intake, classified as either adequate or inadequate based on the nutrient reference value (Estimated Average Requirement) of 100 µg/day for adults, 65 µg/day for children 2–8 years, 75 µg/day for children 9–13 years and 95 µg/day for children 14–18 years [31]; iodine adequacy was used as dichotomous response variable. The logistic regression models adjusted for fish intake (continuous) and, dairy food intake (continuous), socio-economic disadvantage (SEIFA 2011 quintiles [29]), remoteness area (major cities, inner regional and other), and age (in women: 14–18, 19–30, 31–50 years). Results are presented as odds ratios with 95% confidence limits.

In a sub group analysis children aged 5–9 years (*n* = 488), differences in iodine intake between bread intake groups were analysed using the Mann-Whitney U-test for non-parametric data and effect size measured using eta squared (η^2^) statistic (for Kruskal Wallis tests) and Cramers V for chi square analyses. Associations between categories of bread consumption by SES were compared using chi squared analyses.

All analyses were performed using the Statistical Package for Social Sciences (SPSS version 23, Chicago, IL, USA) and *p* values below 0.05 were considered statistically significant.

## 3. Results

### 3.1. Iodine Intake by Age, SES and Geographical Remoteness in the Australian Population (n = 7735)

**Figure 1:**
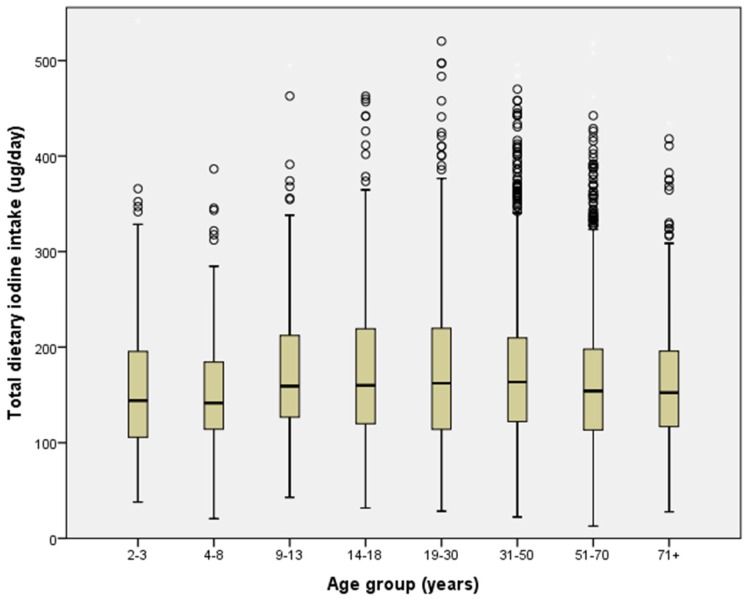
Median dietary iodine intake by age group; 2011–2012 National Nutrition and Physical Activity Survey (*N* = 7735). Error bars show Interquartile Range.

**Figure 2:**
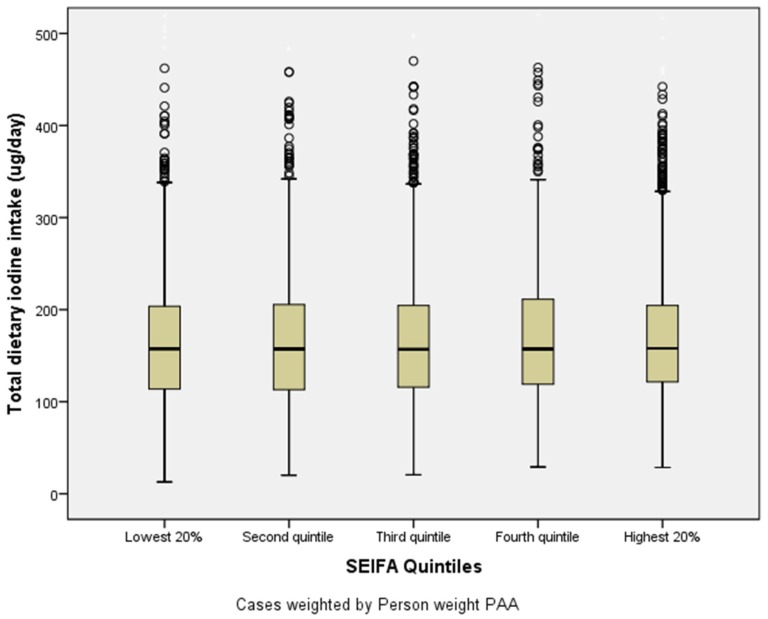
Median dietary iodine intake by socioeconomic status (SES) quintiles; 2011–2012 National Nutrition and Physical Activity Survey (*N* = 7735), Socioeconomic Index For Areas (EIFA) index 2011 of relative socio-economic disadvantage quintile 1 = most disadvantaged, quintile 5 = least disadvantaged [29], Error bars show Interquartile Range.

Median iodine intake differed between age groups (*p* < 0.001, η^2^ = 0.001) (Figure 1), SES quintiles (*p* < 0.001, η^2^ = 0.002) (Figure 2), and geographical remoteness area (*p* < 0.001, η^2^ = 0.000). Adolescents 14–18 years had the highest median iodine intake (177 µg/day), while children 4–8 years had the lowest median iodine intake (152 µg/day) (Figure 1). However, age-related differences were small, with <0.98% difference between groups (η^2^ < 0.0098).

No trend was evident for iodine intake between SES quintiles. Inner regional areas of Australia had slightly higher dietary iodine intakes (mean= 170 µg/day), followed by major cities (mean = 168 µg/day) and then other regions of Australia (mean = 166 µg/day) (*p* < 0.001; η^2^ < 0.002), with less than 0.1% variance in iodine intake explained by age group, SES and location.

### 3.2. Food Group Contributions to Total Iodine Intake in the Australian Population (n = 7735)

The four main sources of iodine for the total Australian population at the time of the survey were: cereals and cereal products (mean 48.1; SD ± 34.5 µg/day, 29%); milk products and dishes (46.7 ± 45.5 µg/day, 26%); non-alcoholic beverages (23.6 ± 21.1 µg/day, 15%) and cereal based products and dishes (19.6 ± 24.4 µg/day, 12%). Smaller contributions were provided by fish and seafood products and dishes (7.1 ± 21.1 µg/day, 4%), egg products and dishes (7.2 ± 15.3 µg/day, 4%), meat, poultry and game products and dishes (4.9 ± 7.2 µg/day, 3%), and vegetable products and dishes (3.7 ± 8.1 µg/day, 2%). In the non-alcoholic beverages group, water provided the greatest contribution (6.6 ± 5.5 µg/day, 38% of food group), followed by coffee and coffee substitutes (8.2 ± 18.4 µg/day, 21%). Within the cereals and cereal products major food group, regular breads/bread rolls contributed the largest amount of iodine (38.1 ± 31.8 µg/day, 72%), followed by English-style muffins, flat breads and savoury and sweet breads (5.1 ± 12.2 µg/day, 10%). The greatest contribution of cereal based products and dishes were from mixed dishes where cereal is the major ingredient (14.1 ± 22.7 µg/day, 49%). Within the fish and seafood products and dishes food group, finfish (2.1 ± 11.6 µg/day, 31%) made the greatest contribution, followed by similar amounts from packed fish/seafood (0.8 ± 5.6 µg/day, 26%) and fish and seafood products (homemade/takeaway) (2.3 ± 12 µg/day, 25%). The greatest contribution of iodine from milk products and dishes was provided by milk (32.2 ± 38.3 µg/day, 62%).

The only difference in food group contribution to iodine according to age was observed for non-alcoholic beverages (*p* < 0.001), in the direction of increased contribution with decreasing age of the women (Table A1). Regarding SES, contribution of iodine from cereals/cereal products (*p* < 0.001) increased with increasing SES, while milk products/dishes showed an increasing contribution in the opposite direction (*p* < 0.001) (Table A2). Those living in areas of higher geographical remoteness had a lower contribution of iodine from fish and seafood products and dishes (*p* < 0.001) and vegetable products and dishes (*p* < 0.001). The most remote areas obtained significantly more iodine from cereals and cereal products (*p* < 0.001), and meat, poultry and game products and dishes (*p* < 0.001) when compared with all inner regional and major cities.

### 3.3. Association between Bread Intake and Adequacy of Iodine Intake in Women of Child-Bearing Age and Children Aged 2–18 Years

At the time of the survey only 8.0% of Australian children aged 2–18 years (*n* = 142/1772) and 8.6% of women of child-bearing age (*n* = 301/3496) reported consuming ≥100 g bread per day. Median bread consumption was 156.5 g/day (IQR 41–71; mean = 57 ± 33 g/day) for children. Women aged 14–50 years had a median intake of 60 g/day (IQR 41–71; mean = 58 ± 35 g/day). A number of logistic regression models were conducted to assess the effect of bread intake on adequacy of iodine intake. The proportion of children who had inadequate iodine intakes below the age-specific EAR was 8.1% (*n* = 144/1772) and was highest in the oldest age groups (5.5%, 8.4%, 7.3% and 14.8%, according to age groups 2–3 years, 4–8 years, 9–13 years, and 14–18 years, respectively; *I*^2^ = 43.52, *p* < 0.001). After adjusting for fish intake (Table 1; Model 2), women of childbearing age consuming ≥100 g bread per day were six times more likely to have an adequate iodine intake than women consuming <100 g bread per day. Inclusion of dairy intake (Table 1; Model 3) reduced the OR slightly but significance of bread intake remained. Adjustment for SES, geographical remoteness area and age (Models 4, 5 and 6) did not further influence the odds of women achieving an adequate iodine intake (i.e., had little predictive value in the model). Similar findings were found for children (Table 2), but a higher OR was found for all the models.

In order to assess whether young children were being exposed to excessive iodine in the food supply, the proportion of children with reported iodine intakes that exceeded the age-specific Upper Level was calculated. Thirty percent of children aged 2–3 years had intakes above the UL of 200 µg/day, while 5.9% of 4–8 years old had intakes above 300 µg/day, while none of the children aged 9–14 years and 15–18 years had intakes above 600 and 900 µg/day, respectively (*I*^2^ = 333.94, *p* < 0.001).

### 3.4. Effect of SES on Bread Consumption Patterns and Association with Iodine Intake in a Subgroup of Children Aged 5–9 Years

Ten percent of Australian children aged 5–9 years (*n* = 50/488) reported consuming ≥100 g bread per day. Children consuming ≥100 g bread per day had a higher median dietary iodine intake of 198 µg/day (IQR 161–238 µg/day) than those consuming <100 g of bread per day (149 µg/day (113–201 µg/day); *p* < 0.001, η^2^ = 0.01). All children consuming ≥100 g exceeded the Estimated Average Requirement [31], with 14% also exceeding the upper limit for their age. The proportion of children consuming ≥100 g bread per day was significantly different between all levels of SES (*p* < 0.01) (Table 3). Children from the middle socio-economic group (quintile 3) were least likely to consume ≥100 g bread per day (7%), as compared with the lowest (15%–16%) and highest (10%–13%) SES groups (*p* < 0.01). The interpretation of Cramer’s V measure of effect size using Cohen’s criteria indicated a relatively weak association between SES and the level of bread consumption (*V* = 0.10).

## 4. Discussion

Analysis of dietary intake data obtained from Australia’s most recent national nutrition survey indicates that iodine fortification of salt used for bread-making has the potential to improve iodine intake. We report that women consuming 100 g or more of bread per day (equivalent to approximately 3 slices) were five times more likely to achieve an adequate iodine intake (EAR = 100 µg/day) compared to those consuming less than this amount, after adjusting for age, dairy intake, fish intake, SES, and geographical location. We also investigated the impact of achieving this bread intake in children aged 2–18 years and identified a much larger magnitude of effect in this age group. Children consuming 100 g or more of bread per day were twelve times more likely to have a dietary iodine intake considered to be adequate. Given these findings, it is noteworthy that our data identified that only 8%–9% of Australian women and children are currently consuming bread at the level that was modelled by Food Standards Australia New Zealand to arrive at the fortificant level of 45 µg iodine per 100 g bread [8]. Given this, the amount of iodine added to bread may need to be adjusted to continue to meet the needs of women, or alternatively additional vehicles for the fortificant may need to be explored. The challenge is to meet the needs of women without exceeding upper levels of iodine intake in very young children, as bread consumption in women and children differed across socio-economic groups and geographical locations within Australia. It is concerning that 30% of the youngest age group of children included in the national dietary survey (2–3 years), had reported iodine intakes that exceeded the age-specific Upper Level of 200 µg/day [31]. Investigation of the contribution of foods to total iodine intake in younger children is warranted in future studies.

Dietary diversification is another potential way to improve dietary iodine intake. As well as fortified bread, we identified that other major sources of iodine for the total Australian population in 2011–2013 included milk products and dishes (26%), non-alcoholic beverages (15%) and cereal based products and dishes (12%). Despite being rich in iodine, fish and seafood contributed only minimally to total iodine intake due to low reported consumption, as has been reported by other studies [23,34]. Previous studies in pregnant Australian women identified that dairy foods were a major contributor to total iodine intake (57%–62%), followed by bread and cereals (19%–21%) with minor contributions from fish and seafood (3%–8%) [14,23].

Pregnant women are most at risk for iodine deficiency because of the increased iodine requirements of both the mother and unborn foetus. However, pregnant women were not adequately sampled in the AHS which is why we have focused the current analyses on women of child-bearing age. Women aged 14–50 years had a mean dietary iodine intake of 155 µg/day, which is only a 9 µg/day increase in mean iodine intake from the 146 µg/day iodine intake estimated from the last national nutrition survey conducted in 1995 [9], despite improvements in food composition data during this time. The fortification program was estimated to increase iodine intake in women of childbearing age by 46 µg/day [8]. Compared to the EAR for pregnancy (160 µg/day), we found that only women consuming ≥100 g of bread per day would be able to meet this, and thereby be adequately protected if they were to become pregnant (and not alter their intakes). It is important to note that the mandatory fortification program was not designed to meet increased requirements during pregnancy and lactation, and instead iodine supplementation has been recommended by the NHMRC [35].

For children, we investigated the impact of bread consumption on iodine intake across the age range of 2–18 years. We also investigated the impact of socioeconomic status on bread and iodine intake in a sub group of children aged 5–9 years. This subgroup was chosen because primary schoolchildren are recommended by the ICCIDD as being the universal reference group for assessing iodine status in populations. Spot urinary iodine concentrations (UIC), expressed as median values, are compared against reference values of 100–199 µg/L as being adequate [36]. Another reason to choose this age group rather than pre-school aged children was guided by the observational data from Tasmania that has demonstrated an association between mild maternal iodine deficiency during pregnancy and reduced educational attainment in children that this is evident at the age of 9 years [37]. Similarly, in the United Kingdom, mild maternal iodine deficiency has been shown to result in decreased IQ in the offspring at the age of 8 years and decreased reading comprehension by age 9 years [38]. It is unknown whether such deficits can be reversed through later iodine supplementation or increased intake. However, three randomised controlled studies support the benefit of an improved iodine status on cognitive functioning in children aged between 6 and 13 years. It is therefore important to identify dietary sources of iodine intake in this age group in order to recommend dietary modifications to address inadequate intakes, for improved health outcomes, if necessary.

The Australian Health Survey collected UIC in a biomedical subsample of the survey, in participants aged 5 years and older. An adequate median UIC was found across all age and sex groups, post fortification [39], with children aged 5–11 years having the highest median UIC (177 µg/L), and only 5.9% of this group having iodine levels under 50 μg/L. This is a major improvement compared to the mild iodine deficiency identified in schoolchildren aged 8–10 years across five mainland states in the Australian National Iodine Nutrition Study, 2003–2004 [2]. The current analysis of dietary iodine intake data allows identification of food sources, including the contribution of fortified bread, to total iodine intake. Additionally, identification of socioeconomic and geographical determinants of iodine intake are useful for dietary guidance purposes and monitoring of the iodine fortification programme.

We found associations with age, SES and geographic remoteness area. However, the small effect sizes of these differences suggest that these factors are of minor consequence. Both women and children in the lowest SES group and those in regional, rather than major cities, had higher bread intakes. This is consistent with findings from New Zealand, which found that low SES pregnant women had a higher iodine intake from bread compared to women in higher SES groups post fortification [18]. Other foods that were found to be more important sources of iodine for high SES groups compared to those in lower quintiles, included fish and seafood, eggs, and vegetables. We hypothesize that this may be due to the high cost of seafood, often making it unaffordable for lower SES groups [16].

These analyses have several limitations. Dietary data collected for the NNPAS consisted of two 24-h recalls, and was adjusted for within person variation by using an average of the two day dietary data. It is well known that repeated 24 h recalls may not be a suitable method for estimating “usual intake” as the method does not account for all sources of error [27]. Under-reporting is a major limitation associated with nutrition surveys using self-reported intake due to a widely observed tendency for people to underestimate their food intakes [40]. In this regard, the contribution of iodised table salt was not assessed in the survey. Australia does not implement universal salt iodisation, but manufacturers are permitted to add iodine to table salt voluntarily [8]. In non-quantitative questions, 29% of NNPAS participants reported using iodised salt during food preparation and 21% reported using iodised salt at the table, thus the current analysis probably underestimates total dietary iodine intake [25]. However, we have previously found that self-reported use of iodised table salt is unreliable, and did not differ between pregnant women who were iodine deficient or iodine replete [3,23].

There is a possibility that the 24-h recall method may have missed foods that are high in iodine but not regularly consumed, such as fish and seafood. A further limitation is that SEIFA, which was used in the survey to report SES, represents an average of all people living in an area; it does not represent the individual situation of each person. Larger areas are more likely to have greater diversity of people and households. However this measure of socioeconomic disadvantage is considered the most appropriate for use in a national survey of this scale [29].

Despite these limitations, the NNPAS data are the most current and comprehensive nationally representative data available on food and nutrient intakes of Australians. This data contributes to evidence regarding the impact of the iodine fortification program and has identified factors associated with group level reported adequate iodine intake on a national level.

## 5. Conclusions

Secondary analysis of national nutrition survey data indicates that the addition of iodine to the food supply through fortification of a staple food vehicle has resulted in adequacy of iodine intake. This is apparent across all socio-economic groups, as well as in residents of remote areas where sources of naturally occurring iodine-rich foods may be less accessible. Fortified bread consumption at the level that was modelled in the fortification program (100 g/day) is associated with a greatly increased odds of achieving an adequate iodine intake in both women of childbearing age and children aged 2–18 years. However, the majority of Australian women and children are not generally consuming bread at this level and therefore ongoing monitoring and surveillance of iodine status in high-risk groups is required.

## Figures and Tables

**Table 1:** Adjusted odds of achieving adequate iodine intake by bread intake for a representative national sample of women of child-bearing age (14–50 years) from the Australian population (*n* = 3496).

	OR (95%CI)	
	<100 g Bread/Day	≥100 g Bread/Day
Model 1 ^1^	Referent	6.04 (5.94–6.13) *
Model 2 ^2^	Referent	5.04 (4.96–5.13) *
Model 3 ^3^	Referent	5.00 (4.92–5.08) *
Model 4 ^4^	Referent	5.01 (4.93–5.09) *
Model 5 ^5^	Referent	5.01 (4.93–5.09) *
Model 6 ^6^	Referent	5.04 (4.96–5.13) *

^1^ Unadjusted model; ^2^ Adjusted for dairy intake; ^3^ Adjusted for dairy and fish intake; ^4^ Adjusted for dairy and fish intake, and Socioeconomic Index For Areas (SEIFA) group [29]; ^5^ Adjusted for dairy and fish intake, SEIFA group and remoteness area [30]; ^6^ Adjusted for dairy and fish intake, SEIFA group, remoteness area and age. * *p*-value < 0.05.

**Table 2:** Adjusted odds of achieving adequate iodine intake by bread intake for a representative national sample of children aged 2–18 years from the Australian population (*n* = 1772).

	OR (95%CI)	
	<100 g Bread/Day	≥100 g Bread/Day
Model 1 ^1^	Referent	13.56 (1.88–97.66) *
Model 2 ^2^	Referent	12.72 (1.76–91.70) *
Model 3 ^3^	Referent	12.86 (1.78–92.74) *
Model 4 ^4^	Referent	12.85 (1.78–92.69) *
Model 5 ^5^	Referent	12.84 (1.78–92.59) *
Model 6 ^6^	Referent	12.34 (1.71–89.26) *

^1^ Unadjusted model; ^2^ Adjusted for dairy intake; ^3^ Adjusted for dairy and fish intake; ^4^ Adjusted for dairy and fish intake, and SEIFA group [29]; ^5^ Adjusted for dairy and fish intake, SEIFA group and remoteness area; ^6^ Adjusted for dairy and fish intake, SEIFA group, remoteness area [30] and age. * *p*-value < 0.05.

**Table 3:** Proportion of Children 5–9 years consuming ≥100 g bread/d by SES quintiles; 2011–2012 National Nutrition and Physical Activity Survey (*n* = 488).

SES Quintile *	1	2	3	4	5
≥100 g/day (*n* = 50)	15.4% ^a^	16.4% ^b^	7.2% ^c^	13.1% ^d^	10.1% ^e^
<100 g/day (*n* = 438)	84.6 ^a^	83.6% ^b^	92.8% ^c^	86.9% ^d^	89.9% ^e^

* SEIFA (Socioeconomic Index For Areas) of relative socio-economic disadvantage in 2011. Quintile 1 = most disadvantaged, quintile 5 = least disadvantaged [29]. Values within a row with different subscript letters were significantly different in post hoc analysis (*p* < 0.05).

## Appendix A

**Table A1:** Dietary iodine intake and food sources of iodine by age group ^#^ in the Australian population; 2011–2012 NNPAS.

Food Group	Percentage Contribution to Iodine Intake																
	2–3 Years		4–8 Years		9–13 Years		14–19 Years		19–30 Years		31–50 Years		51–70 Years		71+ Years		*p*-Value *
	Mean	SD	Mean	SD	Mean	SD	Mean	SD	Mean	SD	Mean	SD	Mean	SD	Mean	SD	
**Non-alcoholic beverages**	15.72 ^a^	12.79	15.34 ^b^	12.74	15.28 ^c^	11.48	14.55 ^d^	10.92	15.51 ^e^	11.42	15.06 ^f^	11.64	14.49 ^g^	10.90	14.47 ^h^	10.96	<0.001 *
Tea	12.36 ^a^	20.97	10.21 ^b^	17.41	11.71 ^c^	19.30	14.68 ^d^	23.48	10.51 ^b^	19.32	13.11 ^f^	21.17	12.16 ^g^	20.72	11.11 ^h^	18.48	<0.001 *
Coffee/coffee substitutes	19.62 ^a^	27.96	22.93 ^b^	30.00	23.73 ^c^	31.18	19.52 ^a^	27.06	23.00 ^e^	29.41	21.06 ^f,^	28.90	20.01 ^g^	29.00	20.13 ^h^	28.32	<0.001 *
Fruit/vegetable juices, and drinks	10.98 ^a^	18.78	11.83 ^b^	21.18	9.14 ^c^	18.26	12.90 ^d^	21.67	11.26 ^e^	20.98	11.34 ^b,^^e^	20.75	12.58 ^g^	22.35	11.89 ^d^	20.40	<0.001 *
Cordials	1.28 ^a^	5.71	0.93 ^b^	5.41	1.72 ^c^	7.10	1.09 ^d^	5.49	1.82 ^e^	8.04	1.31 ^f^	6.06	1.52 ^g^	6.32	1.87 ^h^	8.26	<0.001 *
Soft drinks/flavoured mineral waters	10.34 ^a^	18.53	10.44 ^b^	18.99	13.93 ^c^	21.11	10.65 ^d^	17.63	11.54 ^e^	19.37	11.65 ^f^	19.66	11.61 ^d^	20.23	11.15 ^h^	19.45	<0.001 *
Electrolyte/energy/fortified drinks	0.68 ^a^	5.87	0.86 ^b^	5.59	1.26 ^c^	5.73	0.19 ^d^	1.94	0.72 ^e^	4.53	0.60 ^f^	4.12	0.80 ^g^	5.21	0.79 ^e^	4.97	<0.001 *
Waters	40.87 ^a^	31.28	40.33 ^a^	29.79	35.27 ^c^	27.67	39.13 ^d^	27.84	37.94 ^e^	28.68	38.52 ^f^	28.82	37.89 ^g^	29.46	39.11 ^d^	28.00	<0.001 *
Other beverages	3.87 ^a^	12.60	2.46 ^b^	9.62	3.13 ^c^	12.84	1.82 ^d^	8.72	3.16 ^e^	11.89	2.35 ^f^	10.19	3.43 ^c^	13.06	3.80 ^h^	13.46	<0.001 *
**Cereals/cereal products**	29.09 ^a^	17.45	29.53 ^b^	17.85	28.90 ^c^	17.24	29.71 ^d^	17.78	28.34 ^e^	17.32	29.16 ^f^	17.84	28.74 ^g^	17.65	29.00 ^f^	17.64	<0.001 *
Flours/other cereal grains/starches	4.03 ^a^	14.42	4.14 ^b^	15.55	3.44 ^a,c^	11.91	5.22 ^d^	17.62	4.52 ^e,c^	15.51	5.56 ^e^	17.99	4.67 ^g^	16.84	4.49 ^h^	16.39	<0.001 *
Regular breads/bread rolls (plain/unfilled/untopped varieties)	70.36 ^a^	35.76	72.28 ^b^	36.02	74.47 ^c^	34.16	66.68 ^d^	37.87	69.65 ^e^	37.05	68.84 ^f^	37.48	70.41 ^g^	36.38	69.63 ^h^	36.39	<0.001 *
English-style muffins, flat breads, and savoury and sweet breads	9.25 ^a^	22.00	10.10 ^b^	22.98	8.06 ^c^	20.18	10.63 ^d^	22.78	9.74 ^e^	21.83	10.76 ^f^	23.96	9.49 ^b^	21.99	8.33 ^h^	20.35	<0.001 *
Pasta and pasta products	0.76 ^a^	3.11	1.28 ^b^	8.76	0.85 ^c^	4.88	2.60 ^d^	11.94	1.26 ^e,a^	6.97	1.70 ^f^	9.50	1.84 ^f^	9.56	2.69 ^h^	12.33	<0.001 *
Breakfast cereals, ready to eat	9.55 ^a^	22.60	5.16 ^b^	17.13	4.84 ^c^	15.69	7.94 ^d^	22.29	8.23 ^e,d^	23.08	6.13 ^f^	18.71	5.85 ^g^	17.90	7.84 ^h^	21.41	<0.001 *
Breakfast cereals, hot porridge style	2.43 ^a^	9.48	3.57 ^b^	14.29	4.04 ^c^	16.25	4.01 ^d^	14.69	3.45 ^e^	13.79	4.27 ^f,c^	15.65	3.70 ^g^	13.96	4.61 ^h,f,d^	16.90	<0.001 *
**Cereal based products/dishes**	11.68 ^a^	12.52	12.32 ^b^	15.47	12.26 ^c^	14.04	11.41 ^b^	13.29	11.11 ^e^	12.58	12.16 ^f^	14.05	11.89 ^c^	13.64	11.57 ^h^	13.24	<0.001 *
Sweet biscuits	10.57 ^a^	26.83	11.66 ^b^	26.60	8.65 ^c^	24.00	13.06 ^d^	28.75	10.73 ^e^	26.37	10.36 ^f^	26.39	10.94 ^g,e^	26.93	11.58 ^h^	27.65	<0.001 *
Savoury biscuits	7.67 ^a^	23.82	5.96 ^b^	19.84	4.90 ^c^	17.44	6.21 ^d^	20.46	8.19 ^e^	24.84	6.07 ^f^	20.54	5.28 ^g^	19.53	6.20 ^h^	20.41	<0.001 *
Cakes, muffins, scones, cake-type desserts	14.68 ^a^	29.67	15.84 ^b^	30.92	14.41 ^c,a^	29.04	13.31 ^d^	29.23	12.83 ^e^	28.57	14.06 ^f^	29.25	14.78 ^g^	29.81	17.35 ^h^	32.43	<0.001 *
Pastries	10.50 ^a^	23.72	11.37 ^b^	26.43	9.74 ^c^	24.59	12.45 ^d^	28.80	8.90 ^e^	23.54	9.74 ^f^	25.06	10.40 ^g^	25.67	8.03 ^h^	22.32	<0.001 *
Mixed dishes where cereal is the major ingredient	43.76 ^a^	43.86	38.76 ^b^	42.29	43.46 ^c^	43.86	42.94 ^d^	44.34	44.84 ^e^	44.30	42.30 ^f^	43.90	42.68 ^g^	44.37	41.97 ^h^	44.16	<0.001 *
Batter-based products	2.78 ^a^	13.66	4.14 ^b^	16.28	3.54 ^c^	15.81	0.92 ^d^	7.34	3.49 ^e^	16.16	4.04 ^f^	16.80	3.82 ^f^	16.38	2.10 ^h^	11.42	<0.001 *
**Fish/seafood products/dishes**	3.58 ^a^	9.71	3.21 ^b^	8.14	3.31 ^b^	8.33	3.66 ^c^	9.11	4.67 ^d^	10.58	4.01 ^e^	9.85	3.28 ^f^	8.13	3.77 ^g^	9.85	<0.001 *
Fin fish	8.10 ^a^	26.62	7.03 ^b^	25.12	10.11 ^c^	29.46	10.17 ^d^	29.56	8.48 ^e^	26.46	8.31 ^f^	26.73	8.27 ^g^	26.81	8.60 ^h^	27.33	<0.001 *
Crustacea and molluscs	2.83 ^a,e^	16.52	0.37 ^b^	6.03	1.33 ^c^	11.15	2.01 ^d^	13.22	1.82 ^e^	12.54	2.34 ^a^	14.25	2.61 ^g^	15.19	1.65 ^h^	11.81	<0.001 *
Other sea/freshwater foods	0.61 ^a^	7.76	0.02 ^b^	1.40	0.00 ^b^	0.00	0.98 ^d^	9.82	0.15 ^e^	3.87	0.51 ^f^	6.85	0.21 ^g^	4.26	0.70 ^d^	7.54	<0.001 *
Packed fish/seafood	7.48 ^a^	25.88	7.20 ^b^	25.29	5.79 ^c^	22.82	5.81 ^b^	21.77	9.09 ^e^	28.01	7.70 ^f^	26.10	7.12 ^g^	25.00	5.03 ^h^	21.22	<0.001 *
Fish/seafood products (homemade and takeaway)	6.20 ^a^	23.73	5.28 ^b^	21.90	5.25 ^b^	21.81	6.41 ^d^	23.77	7.80 ^e^	25.87	7.05 ^f^	24.79	6.75 ^g^	24.57	8.85 ^h^	27.80	<0.001 *
Mixed dishes with fish/seafood as the major component	0.53 ^a^	7.04	5.34 ^b^	21.87	2.38 ^c^	14.55	3.49 ^d^	17.74	3.82 ^e^	17.95	1.94 ^f^	13.19	3.04 ^g^	16.51	2.58 ^g^	14.81	<0.001 *
**Egg products/dishes**	4.21 ^a^	7.52	3.37 ^b^	7.56	3.68 ^c^	7.72	3.77 ^d^	7.98	4.38 ^e^	8.51	4.04 ^f^	8.42	4.52 ^g^	9.40	4.59 ^h^	9.62	<0.001 *
**Meat, poultry and game products/dishes**	2.98 ^a^	3.57	3.19 ^b^	4.83	3.13 ^c^	4.74	3.49 ^d^	5.42	3.13 ^e^	4.73	3.27 ^f^	4.61	3.22 ^c^	5.21	2.99 ^d^	3.74	<0.001 *
**Milk products and dishes**	25.93 ^a^	17.89	26.04 ^a^	18.50	26.12 ^b^	17.78	25.63 ^c^	19.30	25.04 ^d^	18.38	25.44 ^e^	18.44	26.21 ^f^	19.05	26.52 ^b^	18.58	<0.001 *
Dairy milk	58.10 ^a^	37.83	58.06 ^b^	37.76	56.98 ^c^	37.10	57.40 ^d^	39.82	59.60 ^c^	38.62	58.38 ^f^	37.96	57.16 ^g^	38.16	60.52 ^h^	37.39	<0.001 *
Yoghurt	10.76 ^a^	23.14	11.08 ^b^	23.63	7.55 ^c^	17.92	7.43 ^d^	19.07	8.42 ^e^	21.01	9.62 ^f^	21.76	7.81 ^c^	19.15	9.37 ^h^	20.50	<0.001 *
Cream	0.61 ^a^	5.68	0.88 ^b^	7.90	0.80 ^c^	7.21	0.62 ^d^	3.91	0.69 ^e^	7.12	0.68 ^f^	5.51	0.60 ^g^	4.91	1.08 ^h^	6.98	<0.001 *
Cheese	9.51 ^a^	18.13	10.49 ^b^	23.14	10.96 ^c^	22.12	11.47 ^d^	23.77	11.95 ^e^	24.57	10.82 ^f^	22.31	12.19 ^g^	24.61	9.53 ^h^	20.65	<0.001 *
Frozen milk products	10.11 ^a^	23.54	7.22 ^b^	18.51	8.64 ^c^	20.08	7.88 ^d^	20.41	7.17 ^e^	19.69	6.86 ^f^	18.12	7.94 ^a^	19.25	7.05 ^h^	19.08	<0.001 *
Custards	0.84 ^a^	7.02	0.27 ^b^	2.98	0.88 ^c^	6.76	0.69 ^d^	5.88	0.49 ^e^	3.62	0.95 ^f^	6.79	1.09 ^g^	7.57	0.57 ^h^	4.67	<0.001 *
Other dishes where milk is the major component	0.34 ^a^	2.48	0.47 ^b^	4.43	0.93 ^a^	5.85	1.26 ^d^	8.79	0.71 ^e^	5.19	1.14 ^f^	7.70	1.19 ^g^	7.36	0.96 ^h^	6.18	<0.001 *
Flavoured milks/milkshakes	4.12 ^a^	16.04	4.08 ^b^	15.66	5.70 ^c^	19.20	4.22 ^d^	16.09	5.27 ^e^	19.05	4.48 ^f^	16.32	5.80 ^g^	19.16	4.75 ^h^	18.14	<0.001 *
**Vegetable products/dishes**	2.25 ^a^	3.03	2.28 ^b^	3.67	2.46 ^c^	3.74	2.64 ^d^	4.99	2.42 ^e^	3.75	2.23 ^f^	3.31	2.72 ^g^	4.28	2.60 ^h^	4.64	<0.001 *
**Other foods ****																	
**Total Iodine intake µg/day**	167.61 ^a^^,^^e^	78.61	169.27 ^b^	74.96	165.62 ^c,a,d^	74.42	165.93 ^d,a,f^	69.17	167.36 ^c,e,f^	74.43	164.02 ^g^	67.82	172.29 ^h^	73.15	174.23 ^i^	82.01	<0.001 *

^#^ Age group categories used by NHMRC for Nutrient Reference Values [31]. * η^2^ < 0.01 indicating low effect size (eta square effect size). ^^^ Using average of two 24-h dietary recalls. ** Includes fruit products and dishes; fats and oils; dairy/meat substitutes; soup; seeds and nut products and dishes; savoury sauces and condiments; legume and pulses products and dishes; snack foods, sugar products and dishes, confectionary and cereal/nut/fruit/seed bars; alcoholic beverages; special dietary foods, miscellaneous, infant formulae and foods. Values within a row with different subscript letters were significantly different in post hoc analysis *p* < 0.05 (adjusted significance).

**Table A2:** Dietary iodine intake and food sources of iodine by SES quintiles ^#^ the Australian population; 2011–2012 NNPAS ^#^.

Food Group	Percentage Contribution to Iodine Intake										
	Quintile 1		Quintile 2		Quintile 3		Quintile 4		Quintile 5		
	Mean	SD	Mean	SD	Mean	SD	Mean	SD	Mean	SD	*p*-Value
**Non-alcoholic beverages**	14.5 ^a^	10.99	15.7 ^b^	11.86	15.2 ^c^	11.58	14.7 ^d^	11.31	14.5 ^e^	11.32	<0.001 *
Tea	11.2 ^a^	19.39	12.0 ^b^	20.03	12.4 ^c^	21.16	12.0 ^b^	20.00	12.8 ^d^	21.01	<0.001 *
Coffee	21.5 ^a^	29.81	20.5 ^b^	28.90	21.2 ^a^	29.42	21.0 ^c^	28.98	21.2 ^d^	28.26	<0.001 *
Fruit and vegetable juices	12.5 ^a^	21.70	11.6 ^b^	20.24	10.6 ^c^	20.39	12.4 ^d^	22.13	11.3 ^e^	20.81	<0.001 *
Cordials	1.2 ^a^	5.85	1.8 ^b^	7.69	1.5 ^c^	6.94	1.3 ^d^	6.13	1.6 ^c^	6.50	<0.001 *
Soft drinks	10.9 ^a^	19.85	11.4 ^b^	18.88	11.5 ^c^	19.05	11.6 ^d^	19.94	12.1 ^bc^	20.42	<0.001 *
Electrolyte/energy drinks	1.0 ^a^	5.93	0.7 ^b^	4.33	0.9 ^c^	5.64	0.4 ^d^	3.26	0.6 ^e^	4.08	<0.001 *
Water	38.5 ^a^	29.95	38.7 ^b^	28.37	38.3 ^c^	28.47	38.6 ^d^	28.71	38.0 ^e^	29.05	<0.001 *
Other beverages	3.3 ^a^	12.23	3.2 ^b^	11.99	3.4 ^c^	13.07	2.6 ^d^	11.01	2.4 ^e^	10.26	<0.001 *
**Cereals and cereal products**	28.2 ^a^	17.98	28.7 ^b^	16.99	28.8 ^c^	17.54	30.3 ^d^	17.79	28.9 ^e^	17.86	<0.001 *
Flours and other cereal grains	5.1 ^a^	17.34	4.8 ^b^	16.82	4.5 ^c^	15.46	4.6 ^d^	16.19	4.9 ^c^	17.03	<0.001 *
Regular breads/bread rolls	68.2 ^a^	38.21	70.4 ^b^	36.36	69.9 ^c^	36.30	71.8 ^d^	35.70	69.5 ^c^	36.93	<0.001 *
English-style muffins, flat breads, and savoury and sweet breads	9.9 ^a^	22.79	10.0 ^b^	22.07	9.7 ^c^	22.63	9.5 ^d^	21.62	9.7 ^e^	22.77	<0.001 *
Pasta	2.4 ^a^	12.43	1.2 ^b^	6.67	1.8 ^c^	8.88	1.5 ^d^	8.71	1.8 ^e^	9.27	<0.001 *
Breakfast cereals, ready to eat	7.2 ^a^	20.39	6.9 ^b^	20.59	6.4 ^c^	18.95	6.6 ^c^	19.64	6.1 ^d^	18.53	<0.001 *
Breakfast cereals, hot porridge style	3.3 ^a^	13.45	3.5 ^b^	13.74	4.5 ^c^	16.23	3.4 ^a^	13.59	4.7 ^d^	16.51	<0.001 *
**Cereal based products and dishes**	12.3 ^a^	14.66	12.0 ^b^	14.20	11.2 ^c^	12.64	11.3 ^d^	12.68	12.3 ^e^	13.99	<0.001 *
Sweet biscuits	10.9 ^a^	26.88	11.3 ^b^	27.23	10.5 ^c^	26.75	10.5 ^d^	25.77	10.9 ^e^	26.77	<0.001 *
Savoury biscuits	6.4 ^a^	20.90	6.6 ^b^	22.06	5.2 ^c^	19.26	6.5 ^d^	21.40	6.1 ^e^	20.40	<0.001 *
Cakes/muffin	14.9 ^a^	30.07	14.0 ^b^	28.55	15.1 ^c^	30.95	13.7 ^d^	29.08	14.9 ^e^	30.11	<0.001 *
Pastries	10.0 ^ad^	25.07	10.4 ^b^	25.95	9.5 ^c^	25.02	9.9 ^ae^	24.51	9.7 ^de^	24.55	<0.001 *
Mixed dishes where cereal is the major ingredient	41.5 ^a^	44.12	42.2 ^b^	43.67	43.1 ^c^	44.20	42.2 ^d^	44.09	43.7 ^e^	44.09	<0.001 *
Batter-based products	3.7 ^a^	16.33	3.2 ^b^	15.07	3.0 ^c^	13.85	4.1 ^d^	17.07	3.3 ^e^	15.18	<0.001 *
**Fish and seafood products and dishes**	3.5 ^a^	8.95	3.4 ^b^	8.79	4.0 ^c^	9.49	4.4 ^d^	10.15	3.6 ^e^	9.20	<0.001 *
Fin fish	8.6 ^a^	27.25	8.1 ^b^	26.65	9.1 ^c^	27.60	7.8 ^d^	26.03	8.8 ^e^	27.53	<0.001 *
Crustacea and molluscs	2.4 ^a^	14.64	1.5 ^b^	11.21	1.5 ^c^	11.21	2.1 ^d^	13.80	2.6 ^e^	15.67	<0.001 *
Other sea and freshwater foods	0.3 ^a^	5.52	0.5 ^b^	6.53	0.0 ^c^	1.43	1.1 ^d^	10.02	0.1 ^a^	2.98	<0.001 *
Packed fish and seafood	6.4 ^a^	23.76	8.3 ^b^	26.96	6.7 ^c^	24.20	7.4 ^d^	25.57	7.0 ^e^	24.88	<0.001 *
Fish and seafood products (homemade and takeaway)	6.9 ^a^	24.52	5.5 ^b^	22.14	6.9 ^c^	24.59	9.0 ^d^	27.94	6.8 ^e^	24.69	<0.001 *
Mixed dishes with fish or seafood as the major component	2.6 ^a^	15.50	2.5 ^b^	14.75	3.4 ^c^	16.95	2.9 ^b^	16.34	2.7 ^d^	15.40	<0.001 *
**Egg products and dishes**	4.0 ^a^	8.58	4.3 ^b^	8.50	4.0 ^c^	7.68	4.3 ^d^	9.07	4.3 ^e^	9.38	<0.001 *
**Meat, poultry and game products and dishes**	3.3 ^a^	4.78	3.3 ^b^	4.29	3.3 ^c^	5.66	3.1 ^d^	4.36	3.0 ^e^	4.47	<0.001 *
**Milk products and dishes**	26.6 ^a^	18.72	25.5 ^b^	18.33	26.4 ^c^	18.70	24.7 ^d^	18.05	25.8 ^e^	19.00	<0.001 *
Dairy milk	60.0 ^a^	37.74	56.8 ^b^	39.09	59.4 ^c^	37.47	58.4 ^d^	37.93	57.2 ^b^	38.09	<0.001 *
Yoghurt	7.6 ^a^	19.14	7.7 ^b^	19.08	9.3 ^c^	21.15	9.2 ^d^	22.12	10.1 ^e^	21.66	<0.001 *
Cream	0.8 ^a^	5.98	0.8 ^b^	6.37	0.6 ^c^	5.74	0.6 ^d^	4.29	0.8 ^e^	6.94	<0.001 *
Cheese	11.4 ^a^	23.36	11.5 ^b^	23.53	10.6 ^c^	22.20	11.0 ^d^	22.82	11.1 ^e^	23.23	<0.001 *
Frozen milk products	7.7 ^a^	20.19	7.6 ^b^	19.30	7.1 ^c^	18.23	7.8 ^d^	19.15	7.4 ^e^	19.27	<0.001 *
Custards	0.9 ^a^	6.56	0.6 ^b^	5.60	0.7 ^c^	5.77	0.6 ^d^	5.21	1.1 ^e^	7.48	<0.001 *
Other dishes where milk or a milk product is the major component	0.6 ^a^	5.08	1.3 ^b^	8.99	1.1 ^c^	7.16	0.9 ^d^	4.90	1.0 ^b^	6.88	<0.001 *
Flavoured milks and milkshakes	4.8 ^a^	17.17	6.3 ^b^	19.81	4.4 ^c^	17.11	4.6 ^d^	17.45	4.8 ^e^	17.09	<0.001 *
**Vegetable products and dishes**	2.3 ^a^	3.72	2.4 ^b^	3.58	2.4 ^c^	3.81	2.7 ^d^	4.87	2.5 ^d^	3.73	<0.001 *
**Other foods ****	5.2 ^a^		4.8 ^b^	20.03	4.6 ^c^		4.5 ^d^		5.1 ^e^		
**Total iodine intake (µg/day)**	169.0 ^a^	75.6	165.4 ^b^	66.3	169.4 ^c^	78.3	173.5 ^d^	76.4	165.2 ^e^	69.1	<0.001 *

^#^ Using average of two 24-h dietary recalls. * η^2^ < 0.01 indicating low effect size(eta square effect size). ** Includes fruit products and dishes; fats and oils; dairy/meat substitutes; soup; seeds and nut products and dishes; savoury sauces and condiments; legume and pulses products and dishes; snack foods, sugar products and dishes, confectionary and cereal/nut/fruit/seed bars; alcoholic beverages; special dietary foods, miscellaneous, infant formulae and foods. Values within a row with different subscript letters were significantly different in post hoc analysis (*p* < 0.05).

## References

[1] Charlton K., Skeaff S. (2011). Iodine fortification: Why, when, what, how, and who?. Curr. Opin. Clin. Nutr. Metab. Care.

[2] Li M., Eastman C.J., Waite K.V., Ma G., Zacharin M.R., Topliss D.J., Harding P.E., Walsh J.P., Ward L.C., Mortimer R.H. (2006). Are Australian children iodine deficient? Results of the Australian national iodine nutrition study. Med. J. Aust..

[3] Charlton K.E., Gemming L., Yeatman H., Ma G. (2010). Suboptimal iodine status of Australian pregnant women reflects poor knowledge and practices related to iodine nutrition. Nutrition.

[4] Hamrosi M.A., Wallace E.M., Riley M.D. (2005). Iodine status in pregnant women living in Melbourne differs by ethnic group. Asia Pac. J. Clin. Nutr..

[5] Burgess J.R., Seal J.A., Stilwell G.M., Reynolds P.J., Taylor E.R., Parameswaran V. (2007). A case for universal salt iodisation to correct iodine deficiency in pregnancy: Another salutary lesson from Tasmania. Med. J. Aust..

[6] Nguyen B.B.D., Southcott E., Potter J., Sneddon A., Hickman P.E. (2010). Iodine deficiency in pregnant women in the ACT. Aust. N. Z. J. Obstet. Gynaecol..

[7] Eastman C.J. (1999). Where has all our iodine gone?. Med. J. Aust..

[8] Food Standards Australia New Zealand (FSANZ) Mandatory Iodine Fortification for Australia. http://www.foodstandards.gov.au/code/proposals/pages/proposalp1003mandato3882.aspx.

[9] Australian Bureau of Statistics 4804.0—National Nutrition Survey: Foods Eaten, Australia, 1995. http://www.abs.gov.au/AUSSTATS/abs@.nsf/0/9A125034802F94CECA2568A9001393CE.

[10] Rahman A., Savige G.S., Deacon N.J., Chesters J.E., Panther B.C. (2011). Urinary iodine deficiency in Gippsland pregnant women: the failure of bread fortification?. Med. J. Aust..

[11] Gallego G., Goodall S., Eastman C.J. (2010). Iodine deficiency in Australia: Is iodine supplementation for pregnant and lactating women warranted?. Med. J. Aust..

[12] Baines J., Cunningham J., Leemhuis C., Hambridge T., Mackerras D. (2011). Risk assessment to underpin food regulatory decisions: An example of public health nutritional epidemiology. Nutrients.

[13] Mackerras D.E.M., Eastman C. (2012). Estimating the iodine supplementation level to recommend for pregnant and breastfeeding women in Australia. Med. J. Aust..

[14] Charlton K., Yeatman H., Brock E., Lucas C., Gemming L., Goodfellow A., Ma G. (2013). Improvement in iodine status of Australian women 3 years after introduction of a mandatory iodine fortification programme. Prev. Med..

[15] Hulshof K.F.A.M., Brussaard J.H., Kruizinga A.G., Telman J., Lowik M.R.H. (2003). Socio-economic status, dietary intake and 10 and 14y trends: The Dutch National Food Consumption Survey. Eur. J. Clin. Nutr..

[16] Darmon N., Drewnowski A. (2008). Does social class predict diet quality?. Am. J. Clin. Nutr..

[17] Konttinen H., Sarlio-Lähteenkorva S., Silventoinen K., Männistö S., Haukkala A. (2013). Socio-economic disparities in the consumption of vegetables, fruit and energy-dense foods: The role of motive priorities. Public Health Nutr..

[18] Mallard S.R., Houghton L.A. (2014). Public health policy to redress iodine insufficiency in pregnant women may widen sociodemographic disparities. Public Health Nutr..

[19] El-mani S., Charlton K.E., Flood V.M., Mullan J. (2013). Limited knowledge about folic acid and iodine nutrition in pregnant women reflected in supplementation practices. Nutr. Diet..

[20] Kettings C., Sinclair A., Voevodin M. (2009). A healthy diet consistent with Australian health recommendations is too expensive for welfare-dependent families. Aust. N. Z. J. Public Health.

[21] Bruner B.G., Chad K.E. (2014). Dietary practices and influences on diet intake among women in a Woodland Cree community. J. Hum. Nutr. Diet..

[22] Baruth M., Sharpe P.A., Parr-Medina D., Wilcox S. (2014). Percieved barriers to exercise and healthy eating among women from disadvantaged neighbourhoods: Results from a focus groups assessment. Women Health.

[23] Charlton K., Yeatman H., Lucas C., Axford S., Gemming L., Houweling F., Goodfellow A., Ma G. (2012). Poor knowledge and practices related to iodine nutrition during pregnancy and lactation in Australian women: Pre-and post-iodine fortification. Nutrients.

[24] Australian Bureau of Statistics 4364.0.55.003—Australian Health Survey: Updated Results 2011-12. http://www.abs.gov.au/ausstats/abs@.nsf/Lookup/4364.0.55.003main+features12011-2012.

[25] Australian Bureau of Statistics 4364.0.55.007—Australian Health Survey: Nutrition First Results—Foods and Nutrients. http://www.abs.gov.au/ausstats/abs@.nsf/Lookup/4364.0.55.007main+features12011-12.

[26] Food Standards Australia New Zealand (2007). AUSNUT—Australian, Food, Supplement and Nutrient Database for Estimation of Population Nutrient Intakes. Canberra. http://www.foodstandards.gov.au/consumerinformation/ausnut2007/.

[27] Food Standards Australia New Zealand (FSANZ) (2016). Assessing the 2011-13 AHS against the Australian Dietary Guidelines—Classification System and Database Development Explanatory Notes.

[28] Australian Government Australia New Zealand Food Standard Code—Standard 2.1.1—Cereals and Cereal Products. https://www.legislation.gov.au/Details/F2014C00030.

[29] Australian Bureau of Statistics 2033.0.55.001—Census of Population and Housing: Socio-Economic Indexes for Areas (SEIFA), Australia, 2011. http://www.abs.gov.au/ausstats/abs@.nsf/mf/2033.0.55.001.

[30] Australian Bureau of Statistics 1270.0.55.005—Australian Statistical Geography Standard (ASGS). Remoteness Structure, July 2011. http://www.abs.gov.au/ausstats/abs@.nsf/mf/1270.0.55.005?OpenDocument.

[31] National Health and Medical Research Council (NHMRC) (2006). Nutrient Reference Values for Australia and New Zealand, Including Recommended Dietary Intakes.

[32] Dunn O.J. (1964). Multiple comparisons using rank sums. Technometrics.

[33] Cohen J. (1988). Statistical Power Analysis for the Behavioural Sciences.

[34] Axford S., Charlton K., Yeatman H., Ma G. (2012). Poor knowledge and dietary practices related to iodine in breastfeeding mothers a year after introduction of mandatory fortification. Nutr. Diet..

[35] National Health and Medical Research Council (NHMRC) Iodine Supplementation during Pregnancy and Lactation. https://www.nhmrc.gov.au/guidelines-publications/new45.

[36] World Health Organization (WHO) (2007). Assessment of Iodine Deficiency Disorders and Monitoring Their Elimination.

[37] Hynes K.L., Otahal P., Hay I., Burgess J.R. (2013). Mild iodine deficiency during pregnancy is associated with reduced educational outcomes in the offspring: 9-year follow-up of the gestational iodine cohort. J. Clin. Endocrinol. Metab..

[38] Bath S.C., Steer C.D., Golding J., Emmett P., Rayman M.P. (2013). Effect of inadequate iodine status in UK pregnant women on cognitive outcomes in their children: Results from the Avon Longitudinal Study of Parents and Children (ALSPAC). Lancet.

[39] Australian Bureau of Statistics 4364.0.55.006—Australian Health Survey: Biomedical Results for Nutrients, 2011-12. http://www.abs.gov.au/ausstats/abs@.nsf/Lookup/4364.0.55.006Chapter1202011-12.

[40] Macdiarmid J., Blundell J. (1998). Assessing dietary intake: Who, what and why of under-reporting. Nutr. Res. Rev..

